# The Role and Value of Chaplains in an Australian Ambulance Service: A Comparative Study of Chaplain and Paramedic Perspectives

**DOI:** 10.1007/s10943-022-01685-4

**Published:** 2022-11-19

**Authors:** Katie Tunks Leach, Paul Simpson, Joanne Lewis, Tracy Levett-Jones

**Affiliations:** 1 Faculty of Health, University of Technology Sydney, Sydney, Australia; 2 New South Wales Ambulance, Sydney, Australia; 3 School of Health Sciences, Western Sydney University, Sydney, Australia; 4 School of Nursing and Health, Avondale University, Wahroonga, Australia

**Keywords:** Paramedic, Emergency medical services, Wellbeing, Chaplaincy, Spiritual care

## Abstract

Chaplains are embedded in several ambulance services across Australia, however as Australia’s religiosity is currently in decline and questions are being asked about retaining chaplains, little is actually known about their role and value within Ambulance services. The aim of this paper is to present the key findings from interviews with chaplains about their role and value of being ambulance chaplains. These findings are then compared with those of paramedics derived from an earlier phase of this study. Thirteen chaplains participated in semi-structured interviews, and data were analysed using framework analysis. The results indicated that ambulance chaplains provided paramedic-centred emotional and spiritual care through proactively and reactively supporting paramedics in their work. Chaplains saw value in their relational approach which facilitated trust and access, did not seek to ‘fix’ or diagnose but instead offered physical and emotional presence, and promoted supportive conversations. Chaplains and paramedics valued operationally trained and equipped ambulance chaplains who provided a relational, around the clock, ‘frontline’ staff support presence in paramedic workplaces, regardless of the paramedic’s personal religious/spiritual beliefs.

## Introduction

Multifaith chaplains are embedded in health and wellbeing teams in many ambulance services across Australia to support the pastoral, spiritual and emotional needs of their staff. It is a role that exists alongside other wellbeing roles including peer support officers and psychologists, as well as exercise and nutrition specialists (Magele, 2020; Mammone & Grant, personal communication, 21 March 2022; NSW Ambulance, 2020b). However, calls for the removal of religious chaplains from some organisations are increasing, especially in Australia where religiosity has been in decline (Kanowski, 2022; Australian Bureau of Statistics, 2022; Caro, 2022; Price, 2022). Subsequent public discourse from those on each end of the for/against spectrum continues, yet notably absent from these discussions are the voices of the people who choose to access chaplain care. As Swinton (2014, p. 172) noted:

“The point that is being made here relates to the importance of recognising and valuing ‘the spiritual’…irrespective of what one thinks its origins might be”.

Studies exploring consumer perspectives of chaplaincy or spiritual care programmes in a range of settings have demonstrated numerous benefits, and in many instances these remain even when the consumer does not share the same personal or religious beliefs as the spiritual care provider (Callis et al., 2021; Department of Education & Training, 2018; Liefbroer & Nagel, 2021). Furthermore, the body of evidence examining the connection between one’s spirituality and health outcomes is significant. For example, positive health benefits for those who are moderately spiritual or religious include reduced burnout and increased longevity and resilience, with these health outcomes reducing and in fact becoming negative for those who are highly spiritual or religious (Birkett, 2015; Frankl, 1985; Koenig, 2012, 2015; Tak et al., 2017). Additionally, research by Tedeschi et al. (2017) demonstrates the essential role of spiritual change and core beliefs in posttraumatic growth (PTG). Another study exploring the role of core beliefs in paramedic Posttraumatic Stress Disorder (PTSD) and PTG further stressed the importance of organisations providing workplace interventions in support of bettering these outcomes (Surgenor et al., 2020). Consequently, more healthcare groups are acknowledging the importance of offering and providing high-quality spiritual care to consumers as part of an holistic approach to enhancing health outcomes (Royal Australian & New Zealand College of Psychiatrists, 2018; World Health Organization, 2021; World Psychiatric Association, 2019).

Despite this evidence, there are limited studies exploring first responder perceptions of chaplains in their services. In a scoping review on this topic, only seven papers were identified across first responder and military settings (Tunks Leach et al., 2020), and since that review, only one additional paper was found that explored consumer perceptions (Flores, 2021). Consequently, in a previous paper, we explored paramedic perspectives on the role and value of chaplains in the ambulance service (Tunks Leach et al., 2022). In the current paper, we present the findings from a subsequent study of chaplains’ views of their role and we compare this with the paramedics’ perspectives, with the aim of creating a comprehensive understanding of the role and value of chaplains in the ambulance service.

## Methods

### Study Design and Setting

Set in New South Wales Ambulance (NSWA) in Australia, this study took place between June and August 2020. NSWA is a state-based ambulance service that employs over 5,900 staff spanning metropolitan, regional and remote areas (NSW Ambulance, 2020a). The chaplaincy service is one of a suite of internal staff support initiatives that include a peer support programme, psychology service, exercise and nutritional support. An external Employee Assistance Program (EAP) is also available (NSW Ambulance, 2020b).

The chaplaincy programme, at the time of this study, employed one paid and 55 volunteer chaplains (McFarlane, personal communication, July 2019). Twenty-three of these chaplains were female and 22 male, with one from the Muslim faith, one from the Jewish faith and 53 from the Christian faith. The ambulance chaplaincy service endeavours to provide 24 h/7-day care to staff, their families, and bystanders, regardless of their spiritual or religious beliefs (NSW Ambulance, 2020b). Chaplains may be activated by a manager or directly by the paramedics for support.

This paper presents the second arm in phase one of an exploratory sequential mixed-methods study, exploring the role of ambulance chaplains and their impact on paramedic wellbeing. It is underpinned by a pragmatic philosophical framework. Driven by the research questions and a commitment to using multiple types of data to answer these questions, a pragmatic framework is a pluralist approach which posits that people will bring multiple viewpoints to the study and are held as being equally true (Biesta, 2010; Creswell & Plano Clark, 2018). This study sought to investigate pastoral, spiritual and emotional care activities undertaken by chaplains and their impact on paramedic wellbeing. This phase of the study comprised semi-structured interviews with NSWA chaplains and, along with the data obtained from the paramedic interviews, the findings will be used to develop a survey that will explore the views of a broader cross-section of paramedics in phases two and three of the study.

In this study, trustworthiness was demonstrated through bracketing, reflexivity and member checking. The first researcher currently practices as a chaplain in NSWA, so bracketing was used prior to undertaking the research so as to document existing beliefs, assumptions and hypotheses (Tufford & Newman, 2010). Reflexive journaling and analytical memos on thoughts relating to participants’ comments, or the author’s own views and observations, were kept throughout the research process (Saldana, 2016). Furthermore, the first researcher also met to discuss findings with the research team at regular meetings. Finally, a summary of the major themes from the qualitative data analysis phase were member checked by sending these themes to the participants for feedback, with nine participants responding to confirm their agreement with the findings (Whitehead et al., 2020).

### Ethical Considerations

South East Sydney Local Health District Human Research Ethics Committee provided primary ethics approval [2019/ETH13593] which was subsequently ratified by the University of Technology Sydney Human Research Ethics Committee [ETH19-3820]. All participants received participant information sheets prior to providing written informed consent, and they were made aware that their responses would be de-identified for privacy and confidentiality, and subsequently used in peer-reviewed publications and presentations.

### Sample and Recruitment

Following ethics approval, participants were recruited via an email sent by NSWA’s research unit and the Senior Chaplain. A purposive sampling approach was used to ensure participants had the required knowledge and experience to answer the research questions, and represented views from different genders and geographical areas (e.g. metropolitan and regional). Follow-up via telephone and email was used to check that participants met the inclusion criteria and were comfortable with the interviewer’s position as a fellow chaplain and researcher (Whitehead et al., 2020). When no new significant themes were emerging, recruitment was discontinued at 13 participants (Coffey et al., 2017; Creswell & Plano Clark, 2018).

### Data Collection

Chaplain interviews took place between June and August 2020. They lasted between 30 and 60 min and were conducted by the first researcher at a mutually agreed location. Interviews were undertaken in-person and over the phone. As they took place in the early stages of the COVID-19 pandemic, decisions regarding how and where interviews were conducted were determined by organisational policies, geographic restrictions (many of the participants resided in regional NSW) and/or participant preferences.

Prior to commencing the interviews, participants were reminded that the researcher’s motivation for conducting the study was to better understand the role of chaplains in the ambulance service and what impact their actions have on paramedic wellbeing. Questions used to guide the semi-structured interviews were generated from a scoping review (Tunks Leach et al., 2020), refined following pilot interviews, and provided to the participants in advance of the meetings (Appendix 1) (Peters et al., 2020). Semi-structured interviews were used to allow the researcher to pursue emerging themes as directed by the participants (Arksey & O'Malley, 2005; Peters et al., 2020). Interviews were audio recorded and transcribed verbatim.

### Data Analysis

Data were analysed using the framework analysis method. Initially developed by Richie and Spencer (1994), this ‘problem focussed’ thematic data analysis approach requires the development of frameworks and matrices, and enables analysis to move beyond purely descriptive to explanatory (Gale et al., 2013; Ward et al., 2013). Transcripts were thematically coded and analysed using the seven steps proposed by Gale et al. (2013), and meetings between researchers occurred at regular intervals throughout this phase to review findings, clarify any issues arising and ensure accuracy of the qualitative methodology. The framework for analysing the chaplain interviews used the same overarching themes as those used to analyse data from the paramedic interviews previously conducted (Tunks Leach et al., 2022); however, the subthemes were specific to the chaplain interviews.

## Results

The final sample consisted of 13 chaplains. Ten participants identified as male and three as female with a mean age of 52 years and duration of service of 5.5 years. Eight chaplains were from Metropolitan Sydney and five from regional NSW. All chaplains were from the Christian faith, self-identifying as either from Evangelical, Pentecostal or Anglican traditions. Three participants had Masters level education, two Bachelors level and four vocational level, with five participants not disclosing their education. This education was not necessarily specific to chaplaincy, rather it included theological, healthcare and management studies. Six chaplains were recognised or ordained by their faith tradition, while seven were employed as chaplains because of other training and/or experience. Four chaplains had previous experience as a paramedic or ambulance officer. Each chaplain was assigned a pseudonym and any identifying features regarding demographics or quotes with personal information were removed to protect confidentiality. In NSW when the interviews were conducted, COVID-19 was not widely circulating in the community. Consequently, most interviews did not explore the impact of COVID-19 on chaplaincy practice.

**Fig. 1:**
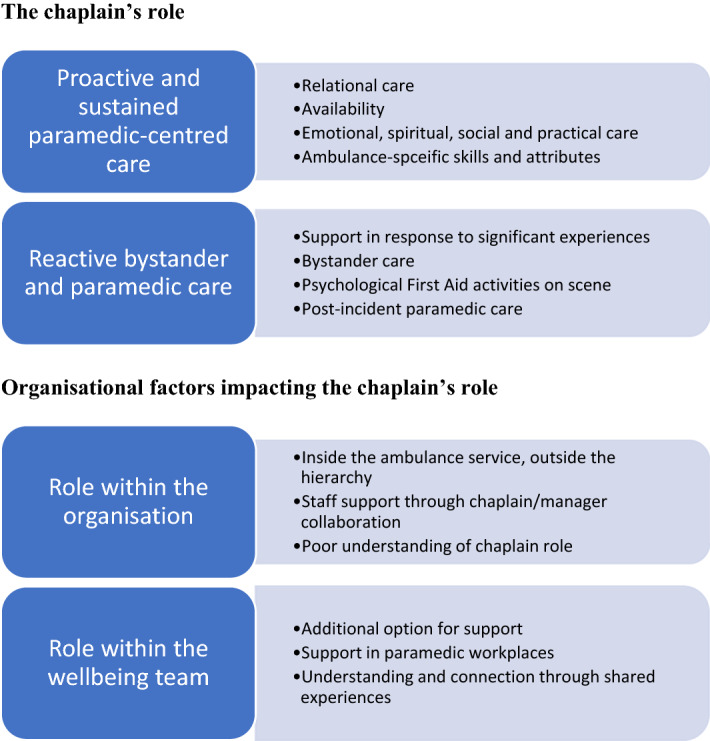
Chaplain results—summary of themes

Findings were grouped into two overarching domains in line with the framework developed for the paramedic interviews: (1) The chaplain’s role and (2) Organisational factors impacting the chaplain’s role (Fig. 1).

### Scope of the Chaplain’s Role

#### Proactive and Sustained Paramedic-Centred Care

Ten of the 13 participants discussed the essential nature of relationships to the chaplain’s role, and they felt that the long-term nature of their role helped to facilitate these relationships and trust. Terms such ‘a friend who cares’ were used to describe the relational element of their role, and how “…it's very different from a psychological paradigm in that you walk with people and…they become your friends, they become family” (Chaplain 9). The participants identified that established relationships underpinned whether paramedics accessed them for support:

“If you haven’t established that relationship, when the big job does come, or the big crisis, or the big family situation, they’re not going to call you. If you haven’t taken the time to go and sit with them on their station and get to know them, you’re not going to be a thought” (Chaplain 6).

Twelve chaplains reflected on the importance of being committed and available, and its impact on building relationships. Maintaining a physical and emotional presence at their ambulance station/s, in and outside business hours, was described as a significant contributor to relationships by ten of the chaplains. Chaplains at “superstations” (stations with more than 100 paramedics) said the volume and turnover of staff was a barrier to building relationships “…because it's a bit of a revolving door there…” (Chaplain 5).

Most chaplains believed their role primarily involved providing pastoral, emotional and spiritual care. This could be related to the job, for example, individual or cumulative job stress and organisational challenges, but also personal support related to relationships, domestic violence, children and general life struggles. This support required chaplains to use active listening skills, and to provide opportunities for the paramedics to reflect and explore their feelings. Chaplains with paramedic experience reflected on the challenges of providing pastoral and emotional care to their colleagues:

“…being a paramedic myself, I know how they work. I know how they internalise things. Sometimes that's what we have to do to try and understand what's just happened…We can be very hard on ourselves as paramedics when stuff goes wrong. It's a very fine road to tread with my fellow officers. [It is] complex” (Chaplain 8).

In addition to emotional care, 11 chaplains spoke about the spiritual support provided in their role. They reflected on conversations involving meaning, purpose, existential explorations, and the ‘big questions’ like life and death:

“The chaplain [addresses] the questions about life and death…about reality, about meaning, fear and hope, stress, loss. Chaplains can explore these issues confidentially, honestly, openly and in that caring loving manner…The goal is to give people meaning and purpose and connection wherever they're at” (Chaplain 3).

Seven chaplains said their role included providing care to meet physical and social needs both to paramedics and their families. Physical care included ensuring paramedics had food, water and shelter, and supporting them with activities like cleaning or restocking ambulances. Social care included exploring and promoting ways for paramedics to connect with recreational and essential services both within and outside of the organisation:

“I think, we’re strongly networked with our community care as well…[a paramedic] said ‘you guys don’t realise how connected you are with so many support services, the rest of us don’t even know about them. Whether its people needing a meal or a roof over their head or something for family violence or whatever, you guys know who to talk to and how to find someone discreetly to help them out, and it’s never a problem’” (Chaplain 1).

When asked about their thoughts on the value of proactive care, chaplains considered: how their relational model of care promoted trust, the value of being a staff support resource that is available around the clock, their ability to provide holistic care that extends beyond the spiritual, and the capacity to care for all staff and not just paramedics.

#### Reactive Support

The second theme identified by chaplains as part of their role was the provision of reactive support. Reactive care took place at or immediately after significant experiences such as a major trauma, natural disaster, terrorist incidents, death by suicide, or death of a baby/child. Reactive care was also provided during and after jobs that held personal significance to a paramedic because of sights, sounds or smells, the cumulative nature of their experiences, or the death of a colleague.

Once on the scene of significant jobs, most chaplains identified that caring for paramedics primarily involved assuming care of bystanders such as family members, witnesses and other first responders including police. Chaplain 12 stated “I supported four policemen, the mother and father of the deceased and the extended family as they filtered in”. Another chaplain stated:

“…sometimes the paramedics call us out because their heart goes out to the family. So, it's not essentially about them, but I guess their compassion extends to the family and they want to make sure that they're okay.” (Chaplain 13).

Care provided by chaplains varied according to the nature of the job. Twelve participants spoke of ‘walking alongside’ paramedics and/or bystanders in the immediate minutes and hours following an incident. This care could be autonomous or manager-directed, but always under the direction of the ambulance commander-in-charge. It included meeting immediate needs (mostly for bystanders but occasionally for paramedics) such as food, shelter and safety. A small number of chaplains spoke of being used as “an extra set of hands” when requested by paramedics. Care also involved explaining police and coronial processes to family members in the aftermath of unexpected deaths, and supporting family members to break bad news to others. After immediate physical and emotional needs were met, chaplain’s reactive care also included providing hope and connecting bystanders to support:

“…it's just gently listening, then moving them to think of their own network, is there anyone that they feel they need to talk to or speak to. Sometimes arranging connections with funeral directors…Especially when the contractors come to take away their beloved. I think just stepping them through that process, but also guiding them emotionally, psychologically and spiritually as well through all of that” (Chaplain 13).

Chaplains providing on-scene support focused their activities on minimising paramedic exposure to strong emotions:

“I've found that the paramedics very much know what they're doing clinically but when there's emotional issues, they will get me involved…I think the paramedics can see where their limitations are but they're also resourced to know that their limitations are someone else's strengths” (Chaplain 12).

Eleven chaplains said observation of paramedics on-scene and immediately after significant jobs was important. Some identified their role was to monitor a scene for threats or areas of need which were possibly not immediately identified by paramedics, while others focused on observing paramedics during clinical debriefs for any who maybe showing signs that “all was not ok”. This was especially true for chaplains with paramedic experience who felt there was additional value in being able to talk about the clinical alongside the emotional:

“…There was one guy in the background who was really quite silent and was avoiding me. He’s somebody I’ve worked with. I spoke to him afterwards and found out that he’d been to three or four paediatric [cardiac] arrests in a couple of weeks…I think it’s something that chaplains look for” (Chaplain 7).

In the hours, days and weeks following significant jobs, most chaplains said they were asked to attend post-incident clinical debriefs and then follow-up with paramedics. A small number of chaplains noted their follow-up care was declined by paramedics on the day of the event, however this was often better received in the days following the job:

“…after a job, paramedics are not real keen to just sit there and have a chat…They just want to get the job done, they want to go and clean-up, they want to go and eat something before someone annoys them with something else. I think being able to recognise when you’re needed, and when you’re not, is a big thing” (Chaplain 6).

### Organisational Factors Influencing the Chaplain’s Role

#### Role Within the Organisation

Nine chaplains stated that being employed within the ambulance organisation (as opposed to being an external provider of chaplain care) had a significant impact on their role. They spoke about the importance of wearing a uniform, undergoing ambulance training and being part of the culture to fully appreciate the pressures on paramedics:

“You need an understanding of emergency services culture…you've got to understand, the shift work, that they're a closed bunch, they're tough. They're exposed to a lot of trauma that the normal population doesn’t see. You've got to have an understanding of the incident control systems, and how they actually function in a job” (Chaplain 3).

While having chaplains retained in the organisation was seen as important, it was equally important to the chaplains that they were outside of the hierarchy:

“A lot of [paramedics] won’t go to their [peer support officers], because the PSOs are now getting in positions of being inspectors and people won’t go to somebody higher than them in the hierarchy” (Chaplain 7).

Being situated outside the hierarchy was perceived to promote trust and confidentiality:

“We're not their partners, we're not their bosses, we have no rank which I think is quite valuable. We sit just outside and offer that human connection and listening ear for everyone, from the seniority down to the guys on the road” (Chaplain 10).

At the same time, the relationship between chaplain and manager was perceived to be important. Some chaplains described their role as being a mediator between management and staff, while others felt their roles were sometimes misunderstood by managers. At times, chaplains were approached by managers for advice and support or to discreetly support paramedics:

“We’re also very good at dealing with the problem child. The thorn in the side of the [Duty Operations Manager (DOM)] that they just can’t deal with because that person’s a pain in their arse. Get the chaplain onto them. All of those things that are not noticed” (Chaplain 6).

Organisationally, chaplains frequently perceived their role to be poorly understood. Six chaplains said people expected them to provide ritual support (e.g. weddings, funerals, baptisms) and moral or ethical advice to the organisation. Eight chaplains believed there was significant misunderstanding about their role more broadly, especially from groups such as the LGBTQI community, having experienced discrimination from some religious organisations. Overwhelmingly, misconceptions related to religion and the role of chaplains:

“When I started as a chaplain, a lot of paramedics were very hesitant…there was kind of an uncertainty of what chaplains do…I think throughout the years people actually see that week-to-week reality of what chaplains do and it's not just a religious thing, we are definitely there to support them and support community members, family members” (Chaplain 13).

Most chaplains were not able to communicate a clear role description, however they could clearly identify key elements of their work. They spoke of conducting informal assessments to determine how they could best support, and also make referrals when an issue was outside their scope of care:

“People come to me and go, can I have a talk to you over a coffee? This is happening, I don’t know what to do, what do you think? It’s kind of at that level and we shouldn’t underestimate the power of listening, but we’ve also got enough experience and professionalism to know when we need to refer onwards and how to refer people onwards too” (Chaplain 1).

Additionally, chaplains said they provided support to paramedics after exams, at court appearances, at graduations and during mass recruitments of staff at the beginning of the COVID-19 pandemic. Skills used by chaplains in the course of their work included basic counselling, knowledge of first aid, grief and loss education and support, marriage preparation, psychological first aid, and knowledge of internal and external referral pathways.

A noteworthy theme resulted from chaplains reflecting on how providing support impacted their own personal wellbeing. They took satisfaction from their “ability to stand in that place where most people don’t want to be, because it’s so emotionally uncomfortable” (Chaplain 1), yet also spoke about how exposure to other people’s emotions meant “sometimes you don’t cope” (Chaplain 7). The impact of these experiences appeared to be short-term with chaplains discussing their self-care strategies, including the use of pastoral supervision, drawing on their personal belief systems and handing over management of jobs to other chaplains. Most reflected on how they also experienced great happiness from their work. Chaplains said they often used these shared experiences as a mechanism for providing better support to paramedics:

“[Paramedics] don't want to unburden something on someone who's not prepared. You're going to hear some horrible things...when you've won their confidence and they know they're in a safe place, it's an open door for them” (Chaplain 8).

When considering the value of chaplaincy to the organisation, chaplains focussed on their ability to be flexible and dynamic. They reflected on their willingness to personalise the support they provided and their ability to fill gaps according to organisational needs:

“Chaplains are now filling the gaps. We’re stepping into spaces that we never had before. [Supporting] 180 paramedic recruits…doing fire deployments, sitting in meetings as a support person…[or getting] a group of chaplains to go to the airport to support the medical staff that are around-the-clock COVID testing. All of these spaces where they don’t know how to fill…we [chaplains] are filling the voids” (Chaplain 6).

While chaplains felt they added value to the organisation, a number stated they did not feel valued. They spoke of the tension between NSWA saying they valued holistic approaches to paramedic health, yet not providing resources or funding to the chaplaincy programme:

“The ambulance as a whole, they verbally support chaplains. I'm not sure how far that would go to helping practically…there was an inspector whose impression was that [ambulance] wants chaplains, but we need to give them more resources in order to help them be effective in their roles” (Chaplain 10).

#### Role Within the Wellbeing Team

Chaplains in NSWA are part of the wider staff health team. Eleven chaplains said their role was to provide another option for staff who needed support, acknowledging that no one service can meet everyone’s needs:

“…organisationally it’s good that they have a number of different things, because not everybody is going to go to a chaplain, not everybody is going to go to a psychologist. People resisted going to psychologists and for years people resisted going to chaplains” (Chaplain 7).

Twelve chaplains spoke about supporting paramedics wherever they worked and not remotely from an office. This work took place at ambulance stations, at hospitals while staff were waiting to offload their patients, or in the ambulances so paramedics or managers could discuss issues in between jobs. For regional chaplains, this was perceived to be even more important due to the lack of staff support roles in regional areas:

“…my local peer support officer is three hours away…the DOM might be a couple of hours away…for regional areas, the chaplain provides face-to-face contact in an area where you may not see anybody... When the chaplain comes in they sit and have a coffee or a chat, or take you up the street for a coffee so you can get out of the station and just talk to somebody that cares about you” (Chaplain 6).

Most chaplains believed they added value as part of the wellbeing team, especially through “pastoral” care. They spoke of the value in being perceived as neutral and not seeking to make a diagnosis, fix things or critique clinical performance. They saw their role as listening and being present with paramedics through their experiences. Eight chaplains used the words ‘journey’ and ‘alongside’ to explain the value of their work in connection with the previous themes of relationship, geographical location of chaplain work and shared experiences with paramedics. They believed it not only helped them tailor their care, but also gave them credibility with paramedics for their willingness to be “in the trenches” at any time of the day or night:

“He said ‘You're not just an outsourced support network like an EAP psychologist who doesn't understand our context, but you actually are with us and you look like us. More than that, you ride along with us. You actually do the hard yards with us. It's not just about big jobs, you're doing life with us’” (Chaplain 13; relaying feedback from a paramedic).

While chaplains spoke of their role in addressing the “big questions”, more than half spoke about how “having a holistic approach to [paramedic] wellbeing, including their spiritual wellbeing, actually makes them better at what they do on the frontline (Chaplain 1)”.

However, some chaplains felt that because they were volunteers, their worth was less than that of paid support staff:

“I know they [staff health] keep saying that we [chaplains] are an integral part of it, and we work together, but I think they see themselves as a separate entity and we're just around to fill in the holes” (Chaplain 5).

## Discussion

The aim of collecting data from the chaplains in addition to paramedics was to obtain a ‘360 degree’ perspective of the chaplain role, and to see if any new data could be obtained that would address the research aims. Where paramedic interviews gave a broad perspective on the chaplain’s role and its value, the addition of the chaplain interviews added depth and more detailed explanations of chaplain activities.

In this study the activities outlined by chaplains painted a picture of a proactive and reactive frontline role, providing holistic care to paramedics and psychological first aid to bystanders. Chaplains undertaking these activities used skills including assessment, support, counselling, education and ritual in line with the spiritual interventions outlined by the World Health Organization (Carey & Cohen, 2015; WHO, 2017). These findings were common to both paramedics and chaplains, along with a broader understanding that chaplains provided emotional support, social connection and practical or pastoral support. These findings also align with broader research regarding chaplaincy utility and their pastoral/spiritual care activities (Carey, 2012; Carey & Rumbold, 2015; Fitchett, 2017; Liberman et al., 2020).

A major theme discussed by the chaplains was the provision of spiritual care, supporting paramedics with existential, religious and ‘big life questions’. However, this was only a minor theme in paramedic interviews. Reasons for this are not clear, but factors may include a lack of shared understanding of what constitutes spiritual care (i.e. activities paramedics do not see as spiritual, yet chaplains do), chaplains over-reporting or paramedics under-reporting spiritual care encounters, stigma surrounding help-seeking behaviours, or spiritual conversations being deeply personal and paramedics were therefore reluctant to discuss them with the researchers (Morgan et al., 2016; Zullig et al., 2014).

Chaplains providing holistic care, which incorporates not only spiritual but also bio-psycho-social-emotional approaches, align with findings from Spiritual Health Australia on what constitutes high-quality spiritual care (Spiritual Health Association, 2020). Connections between these elements of holistic person-centred care and common health phenomena experienced by paramedics, require further exploration in the light of this study. For example, the provision of emotional and social support promotes PTG, a far more common outcome from exposure to traumatic events than PTSD (Tedeschi et al., 2017). The management of moral injury (MI), a condition recognised as being experienced by paramedics exposed to morally injurious events, is increasingly involving chaplains as part of an holistic healthcare approach. (Koenig & Al Zaben, 2021; Murray, 2019). In fact, emerging research in the military MI space is demonstrating the value of chaplain-lead and chaplain-psychology collaborative models of care in promoting recovery from MI (Ames et al., 2021; Carey & Hodgson, 2018; Hodgson et al., 2021; Koenig & Al Zaben, 2021).

From the chaplains’ perspective, their role centred around and was valued because of its relational model of care. Because chaplains endeavoured to be available 24 h/7 days to share experiences alongside paramedics in their ‘down times’ and ‘on the job’, chaplains felt this facilitated familiarity and trust with the people they supported. This correlated with the paramedics’ perspectives and other healthcare chaplaincy literature (Aiken, 2022; Cunningham et al., 2017). However some paramedics stated that chaplains were mostly only volunteers, which acted as a barrier when they were considering using chaplains as a support resource (Tunks Leach et al., 2022). Yet, when chaplaincy is considered through a healthcare lens, the work of chaplains sits under a primary health care banner (World Health Organization, 2022) and should be more available to paramedics simply because chaplains provide person-centred care in the workplace. This can empower paramedics to take control of their wellbeing and faciliate their access to further treatment when required.

Challenges however clearly remain for ambulance chaplains. Chaplain and paramedic interviews confirmed that ambiguity remains in relation to chaplain activities. A number of paramedics stated they were not clear on exactly what chaplains do, and voiced concerns about the religious association with the ‘chaplain’ title/symbolism. However most said this was overcome through the development of relationships. This finding regard the ‘chaplain' title however was not mirrored in the chaplain interviews. While some chaplains identified wider societal concerns regarding the chaplain's role and religion, the majority did not specifically discuss the use of the word chaplain or the associated symbolism on their uniforms, only stating they would not discuss religion unless specifically requested. Confusion surrounding the chaplain’s role is not new in the literature and suggests a problem with role clarity more globally (Best et al., 2021; Layson et al., 2022; Pater et al., 2021). Further studies are required to determine if this in fact prevents paramedics from accessing chaplains as a support option.

Exploration of the chaplain role on a national and global level provides more clarity on why these challenges may exist at the local ambulance level. Lack of consistent and standardised education pathways are impacting on professionalisation of spiritual care practitioners in Australia. Furthermore, only two professional organisations have developed standards of practice for spiritual care practitioners, and there is currently no mandate for membership to either of these organisations (Spiritual Care Australia, 2020; Spiritual Health Association, 2022). Clinical Pastoral Education, seen as a mainstay of chaplain credentialling in many countries around the globe, is also not mandatory for chaplaincy practice in Australia. Consideration should be made whether chaplains would benefit from working towards recognition with the Australian Health Practitioner Regulation Agency (AHPRA) which would serve to professionalise spiritual care practitioners, provide clarity to other health care workers on the role of spiritual care, and provide protection to the consumers of chaplaincy care (Australian Health Practitioner Regulation Agency, 2022). These challenges with chaplaincy education and professional identity are actively being examined by researchers both in Australia and abroad (Cadge et al., 2019, 2020; Holmes, 2021; Swinton, 2013).

## Limitations

This study into ambulance chaplains contained several limitations. The most significant was the lack of diversity, with most chaplains in this study representing white Christian male perspectives. The lack of diverse faith group representation may be because the chaplaincy service in this study only employs one Muslim and one Jewish chaplain, and electing to participate in this study makes anonymity challenging. Furthermore, there were only participants from one ambulance service in Australia. Exploration of chaplaincy in other ambulance organisations is important to see if this lack of diversity or other findings can be generalised to ambulance chaplaincy programmes more widely.

## Conclusion

While some contemporary discourse may argue for the removal of chaplaincy, phase one of this study has highlighted the critical importance of not confusing ‘popular opinion’ with evidence, and instead adopting a person-centred approach that seeks the opinions of chaplaincy providers and consumers. Participants in this study believed uniformed and operationally capable ambulance chaplains can provide paramedic-centred pastoral, emotional and spiritual support through proactively and reactively working alongside paramedics and sharing their experiences in all the places where they operate. Chaplains and paramedics valued this care because of its relational approach which: (1) facilitated trust, (2) did not seek to fix or diagnose but instead offer physical and emotional presence, (3) sought to be available at any hour of the day or night, (4) reduced barriers to help-seeking and (5) promoted engagement in supportive conversations that facilitated referrals to additional support when required. Overlaying paramedic perspectives on these findings, suggests these views exist regardless of the paramedic’s personal spiritual beliefs. Further work is required to identify if these findings are generalisable to the wider Australian paramedicine context. Additonal research is also needed to explore the connection between the provision of chaplaincy care and health experiences, such as posttraumatic growth and moral injury, and the need to further professionalise the chaplain’s role in Australia.

## Data Availability

Requests for access to the framework matrices can be made to the first author.

## Appendix 1: Interview Questions

The following questions were used to guide the semi-structured interviews. They may or may not have been asked in the order listed, depending on the flow of the conversation:

1. What do you understand the chaplain’s role to be?
2. What skills and attributes should chaplains possess to be effective?
3. Do you think chaplains are valuable to the organisation? If so, how?
4. Do you think chaplains are valuable to staff individually? If so, how?
5. Do you think interactions with chaplains impact staff wellbeing? If so, how?
6. Is there anything else you would like to tell me more about in relation to chaplains, or anything else we have discussed today? This may include a story or an observation, or anything you wish to add.
